# Baduanjin’s impact on quality of life and sleep quality in breast cancer survivors receiving aromatase inhibitor therapy: a randomized controlled trial

**DOI:** 10.3389/fonc.2022.807531

**Published:** 2022-08-05

**Authors:** Jingwen Liao, Yanyu Chen, Li Cai, Kun Wang, Shenghui Wu, Lan Wu, Bixiao Song, Min Hu, Xiaohui Hou

**Affiliations:** ^1^ Guangdong Provincial Key Laboratory of Physical Activity and Health Promotion, Guangzhou Sport University, Guangzhou, China; ^2^ Scientific Research Center, Guangzhou Sport University, Guangzhou, China; ^3^ Department of Sports and Health, Guangzhou Sport University, Guangzhou, China; ^4^ Department of Martial Arts, Guangzhou Sport University, Guangzhou, China; ^5^ Department of Breast Cancer, Cancer Center, Guangdong Provincial People’s General Hospital, Guangdong Academy of Medical Sciences, Guangzhou, China; ^6^ Department of Epidemiology and Biostatistics, University of Texas Health San Antonio, Laredo, TX, United States; ^7^ School of Kinesiology, Shanghai University of Sport, Shanghai, China; ^8^ The Sports and Health Promotion International Collaboration and Innovation Center for People with Disability, Guangzhou, China

**Keywords:** breast cancer survivors, quality of life, sleep quality, aromatase inhibitor therapy, Baduanjin exercise

## Abstract

**Purpose:**

The aim of the current study is to investigate the impact of Baduanjin, a traditional Chinese exercise, on quality of life and sleep quality in breast cancer survivors receiving aromatase inhibitor (AI) therapy.

**Methods:**

A total of 72 breast cancer survivors who had received AI treatment for more3 than 6 months were enrolled in the current study using non-probability consecutive sampling procedure. Participants were randomly assigned in a 1:1 ratio to a 12-week Baduanjin exercise program or to a wait-list control group. The Baduanjin exercise group performed two 90-min supervised sessions per week. The primary outcomes were changes in quality of life measured by the European Organization for Research and Treatment of Cancer Quality-of-Life Questionnaire Core 30 (EORTC QLQ-C30) and in sleep quality evaluated using the Pittsburgh Sleep Quality Index (PSQI).

**Results:**

A total of 68 participants completed the trial (Baduanjin exercise group: n = 33; control group: n = 35). Baseline values for quality of life did not differ between groups. Both global quality of life and physical functioning scores increased significantly by 12.39 (P < 0.001) and 8.48 (P < 0.001) in the Baduanjin exercise group compared with those in the control. Overall PSQI score also decreased by 4.85 (P < 0.001) in the Baduanjin exercise group, whereas it increased by 0.34 in the control group.

**Conclusion:**

Baduanjin exercise training led to improvement in the quality of life and sleep quality of breast cancer patients undergoing AI therapy.

## Introduction

Breast cancer is the most diagnosed neoplasm and the most represented cause of death in women worldwide (1). Aromatase inhibitors (AIs), a form of endocrine therapy, are a mainstay of the adjuvant approach for reducing the growth-stimulatory effects of estrogen in hormone-positive breast cancer of postmenopausal women (2). However, AIs would cause side effects including myalgia, arthralgia, and fatigue that may lead to medication non-adherence and significant decrease in quality of life (3, 4). Furthermore, there is evidence in the literature that approximately 20%–70% of breast cancer patients suffer from poor sleep quality (5). Due to these adverse impacts, adherence to AI therapy is poor (6, 7). Furthermore, long-term cancer treatment imposes an economic burden on cancer patients and subsequently reduces their quality of life (8, 9). Therefore, it is important to develop effective and affordable treatment strategies to improve quality of life and sleep quality for breast cancer patients undergoing AI therapy.

The effectiveness of exercise interventions on alleviating the negative effects of treatments in breast cancer patients and survivors has been well reviewed (10). More recent evidence supports that both resistant exercise (11) and mixed protocols (including sessions of aerobic and strength training) (12, 13) are safe and effective in muscle function, physical performance, and quality of life among breast cancer patients with AI treatment. An interesting type of physical exercise (compared to established types such as aerobic exercise) that might be beneficial for breast cancer patients is Baduanjin (14), a traditional Chinese mind–body exercise incorporating and combining different slow-motions and breathing exercises (15). One advantage of Baduanjin is that it is based on eight simple movements that can be easily learned and are derived from Chinese medical theory (16). On the other hand, Baduanjin has been reported to be safe to perform with relatively few adverse events (17). The beneficial effects of Baduanjin have been fully reviewed; it can improve cognitive function (15, 18), cardiopulmonary function (19), and mental illness (20). Even though there are studies reporting that the Baduanjin exercise had positive clinical effects on breast cancer patients (including quality of life and sleep quality) (21, 22), few indicated the use of AIs. It is currently not well-investigated whether the Baduanjin exercise is an effective way to improve self-reported quality of life and sleep quality in breast cancer survivors undergoing AI treatment. Thus, the aim of the current study is to examine the impact of Baduanjin on measures of self-reported quality of life and sleep quality in breast cancer patients undergoing AI therapy.

## Materials and methods

### Study design and recruitment

This single-blinded randomized controlled trial was conducted in Guangdong Provincial People’s Hospital at baseline and 12 weeks after the treatment using consecutive sampling. This non-probability sample selection for the recruitment is a rigorous process of conducting research including those who meet the predefined inclusion and exclusion requirements by the residents. Therefore, every breast cancer survivor staying in the hospital had an equal chance of being recruited as a participant in this study. Eligible participants met the following criteria: 1) age between 18 and 75 years; 2) diagnosis with stage I–III breast cancer for 6 months to 8 years prior to recruitment; 3) undergoing AI treatment for more than 6 months; and 4) never participated in Baduanjin exercise training in the last 6 months or no previous Baduanjin training experience for more than 3 months. The following exclusion criteria were used: 1) unstable or serious neurologic disease, musculoskeletal disorder, renal failure, cardiovascular or respiratory diseases such as uncontrolled hypertension, and chronic obstructive pulmonary disease; 2) plan of a surgery, such as a joint replacement for the next 6 months; or 3) high-intensity physical exercise experience more than 5 h per week. The ethics committee of Guangdong Provincial People’s General Hospital [Approval No. GDREC2016424H (R1)] approved this research protocol, and each participant has provided written informed consent. This trial was registered in ClinicalTrials.gov PRS (NCT03162133), and all procedures were in accordance with the principles stated in the Declaration of Helsinki.

### Baduanjin intervention

**Figure 1:**
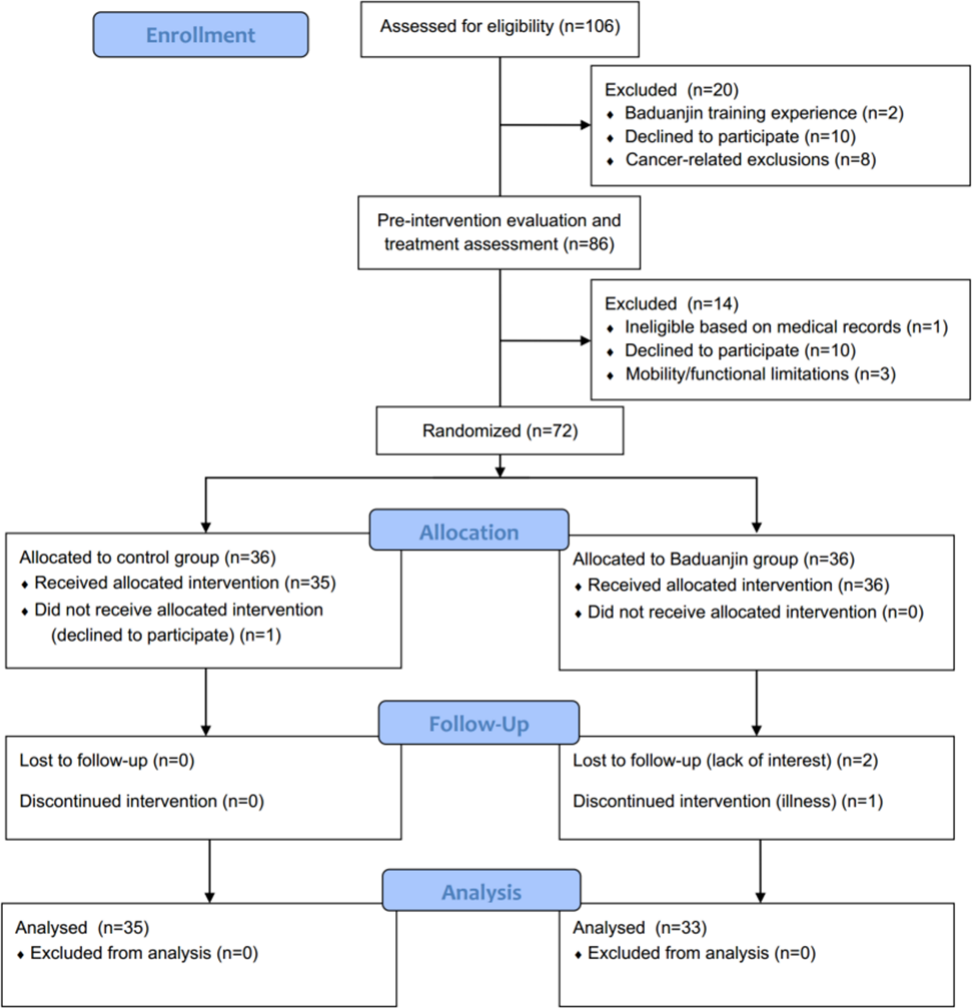
Flow Diagram Depicting the Study Design.

**Figure 2:**
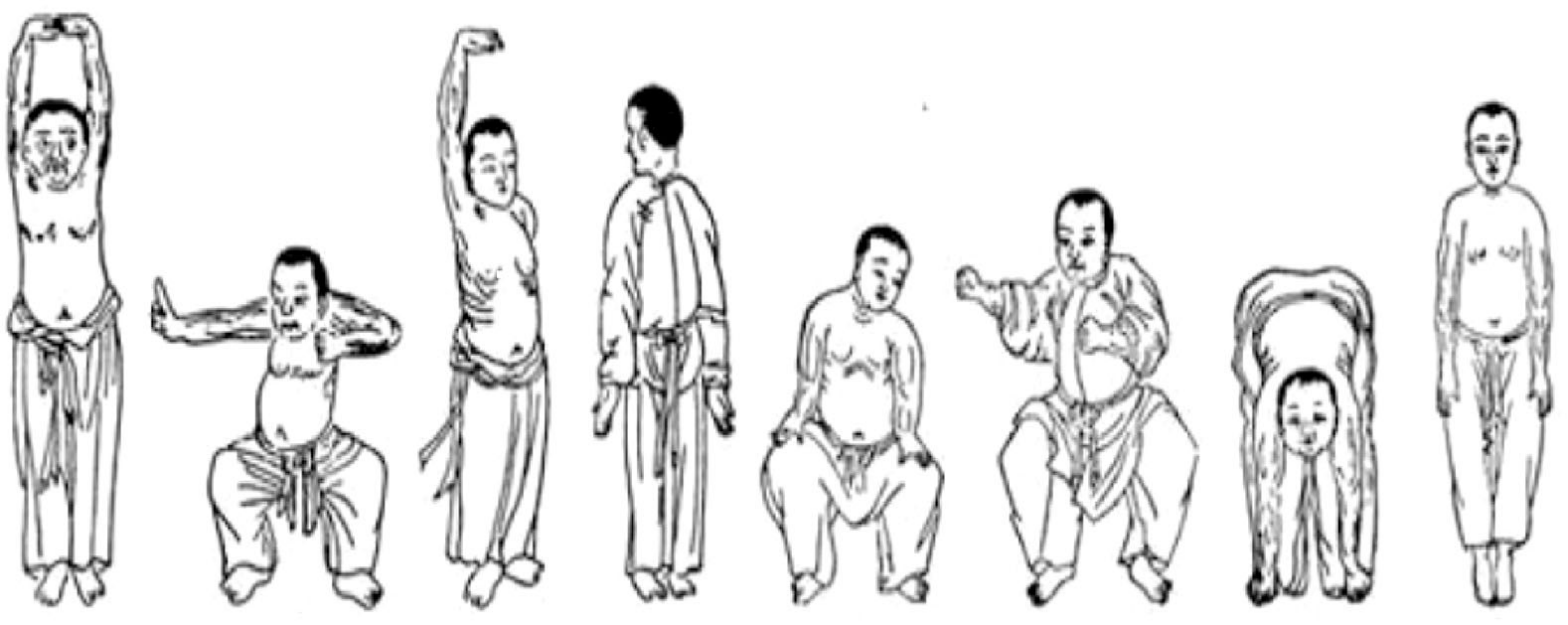
Eight Postures of the Baduanjin Form.

Eligible participants were allocated either to a 12-week Baduanjin intervention group or to a wait-list control group (**Figure 1**). After baseline tests, participants of the Baduanjin intervention group were instructed to attend a Baduanjin program. The program was composed of 90 min per session with 2 sessions per week (Monday and Wednesday) for 12 weeks. Each session consisted of a 10-min warm-up, a 70-min Baduanjin form, and a 10-min cooldown. The whole Baduanjin form included eight postures and was in accordance with the standardized Baduanjin training program “Health Qigong Baduanjin Standard” established by the General Administration of Sports of China (23). Briefly, the 8 postures were (as shown in **Figure 2**): 1) prop both hands to the sky; 2) draw a bow on both sides like shooting a vulture; 3) raise a single arm; 4) look back; 5) sway the head and shake the tail; 6) clench fists; 7) touch toes by hands with flexion of hip and extension of knee joint; 8) rise and bounce on the toes seven times. Two senior Baduanjin instructors from Guangzhou Sports University conducted the training and recorded the participants’ attendance. Participants of the wait-list control group were instructed to continue performing their usual care and daily activities and to refrain from doing any Baduanjin exercise. After their posttest, they were able to attend the Baduanjin program.

### Outcome measurements

#### Quality of life: European organization for research and treatment of cancer quality-of-life questionnaire core 30

The primary outcome was quality of life measured by European Organization for Research and Treatment of Cancer Quality-of-Life Questionnaire Core 30 (EORTC QLQ-C30), which is a 30-item scale that are grouped into the following: five function scales (physical, role, emotional, cognitive, and social), nine symptom scales (fatigue, nausea/vomiting, pain, dyspnea, insomnia, appetite loss, constipation, diarrhea, and financial difficulties), and a global score of quality of life. All scales were linearly transformed to a 0–100 score according to the EORTC Scoring Manual. A higher score for functional scales and global quality of life reflects a better level of functioning, while a higher score in symptom scales indicates a high level of problems (24).

#### Sleep quality: Pittsburgh sleep quality index

The Pittsburgh Sleep Quality Index (PSQI) was used to assess sleep quality at baseline an d post-intervention. The questionnaire consists of 19 questions, and seven component scores were then generated: sleep quality, sleep latency, sleep duration, sleep efficiency, sleep disturbances, use of sleeping medication, and daytime dysfunction. Scoring of answer was based on a scale of 0–3 (total score of 21). A global score was calculated from summing the subscale scores. Lower scores indicate better sleep quality.

### Demographics and medical history

Demographic data (name, age, weight, height, marital status, and employment status) were collected by interviewing the participants. Medical history (time since diagnosis, time since initiating AI, breast cancer stage, type of surgery, comorbidities, and complications) was obtained from medical records.

### Sample size

The population of the current study included individuals diagnosed with breast cancer. In order to calculate the sample size, power was assumed as 0.90 and type 1 error as 0.05. Considering a 20% attrition rate, a sample size of 72 participants for the two groups was finally decided to be needed. Power in changes of quality of life (EORTC QLQ-C30) was calculated to determine the effect size (d) of the Baduanjin intervention. Power in global quality of life was 0.999 (d = 0.704); the highest power was found in Physical functioning by 0.999 (d = 0.746) and lowest in Financial by 0.055 (d = 0.005). Effect size is interpreted as d = 0.20 (small), d = 0.50 (medium), and d = 0.80 (large).

### Randomization and blinding

After baseline assessment, 72 patients who met the inclusion and exclusion criteria were allocated by simple randomization with 1:1 ratio (control group:exercise group) performed by the random number table. Allocation concealment was conducted by the envelope method. Due to the obviously different programs between groups, this study is a single-blinded study. The outcome measurements were evaluated by trained assessors who were unaware of group allocation; the participant was required not to talk about the received intervention.

### Statistical analyses

SPSS 17.0 (IBM, Armonk, NY, USA) was used to perform all statistical tests. Descriptive analyses were applied to characterize baseline patients; differences between groups were compared using independent-samples t-test for continuous data and chi-square test for categorical data. The primary outcomes (scores of the EORTC QLQ-C30 or PSQI) between groups of baseline, post-intervention, or the changes used independent-samples t-test. Intragroup differences were analyzed using paired t-test. Analysis of the outcomes was performed based on the intention-to-treat principle. Minimal detectable change at 90% confidence (MDC_90_) was calculated to evaluate the efficacy of the Baduanjin training on outcome measures. The level of significance was set for all statistical tests at P < 0.05.

## Results

### Flow of participants through the trial and baseline characteristics

Participants were recruited from the Breast Surgery Clinic of Guangdong Provincial People’s General Hospital from November 2016 to May 2017 using a non-probability consecutive sampling. Among the 72 patients who met the enrollment criteria and were willing to participate, 38 participants completed the trials and were included in the analysis. **Figure 1** shows the flow of participants throughout the trial. **Table 1** presents the baseline characteristics.

**Table 1:** Baseline characteristics of patients*.

Characteristics	Control group (n = 35)	Exercise group (n = 33)	P^#^
Age, years	54.63 (8.44)	53.12 (7.02)	0.905
BMI, kg/m^2^	23.37 (3.92)	22.14 (2.67)	0.450
Marital status, No. (%)			
Married	31 (88.6)	22 (66.7)	0.058
Divorced/separated	1 (2.9)	0 (0)	
Single	0 (0)	2 (6.1)	
Unclear	3 (8.6)	9 (27.3)	
Employment status, No. (%)			
Employed full- or part-time	16 (45.7)	14 (42.4)	0.530
Unemployed	3 (8.6)	3 (9.1)	
Retired	14 (40.0)	16 (48.5)	
Unclear	2 (5.7)	0 (0)	
Time since diagnosis, years	2.17 (2.13)	3.00 (2.54)	0.244
Time since initiating AI, years	6.66 (2.27)	2.40 (2.31)	0.195
Overall grade, No. (%)			
I	10 (28.6)	5 (15.2)	0.548
II	16 (45.7)	20 (60.6)	
III	8 (22.9)	7 (21.2)	
Missing/NA	1 (2.9)	1 (0)	

BMI, body mass index; SD, standard deviation; AI, aromatase inhibitor.

*Data are presented as the mean (SD) for continuous variables and frequency (percentage) for categorical variables.

^#^P value for difference between groups.

### Outcomes and estimation

#### Changes in quality of life

**Table 2** summarizes the quality of life outcomes based on scores of the EORTC QLQ-C30. The baseline scores did not differ between the two groups. After 12 weeks of intervention, significant changes from baseline were observed for most functional scales, some symptom scales, and global score (P < 0.05). Functional scales increased significantly, among which physical functioning score increased by 8.48 in the exercise group while it decreased by 3.66 in the control (P < 0.01). For symptom scales, significant decreases in the Baduanjin exercise group were also observed for fatigue (P = 0.010), nausea/vomiting (P = 0.047), pain (P = 0.014), insomnia (P = 0.020), and diarrhea (P = 0.038) compared with those in the control.

**Table 2:** Effects of Baduanjin on quality of life (EORTC QLQ-C30)*.

	Baseline		P^#^	Post-intervention		P^#^	Change		P^&^
	Control	Exercise		Control	Exercise		Control	Exercise	
Physical functioning	85.31 ± 14.84	82.91 ± 12.03	0.261	81.66 ± 13.35	91.39 ± 7.58	0.513	-3.66 ± 13.78	8.48 ± 8.65	0.000
Role functioning	89.97 ± 16.77	85.45 ± 18.98	0.251	88.69 ± 16.49	92.33 ± 10.81	0.869	-1.29 ± 14.64	6.88 ± 17.66	0.015
Emotional functioning	78.29 ± 13.95	75.52 ± 18.72	0.700	78.06 ± 13.45	84.94 ± 13.39	0.330	-0.23 ± 12.36	9.42 ± 19.15	0.010
Cognitive functioning	79.91 ± 17.94	75.64 ± 20.8	0.440	79.09 ± 15.80	84.85 ± 10.79	0.882	-0.83 ± 17.00	9.21 ± 21.42	0.026
Social functioning	82.37 ± 20.57	78.21 ± 29.03	0.834	84.8 ± 18.61	87.73 ± 17.61	0.121	2.43 ± 18.29	9.52 ± 24.72	0.193
Fatigue	32.23 ± 23.61	31.12 ± 19.88	0.789	27.49 ± 22.16	14.24 ± 12.47	0.855	-4.74 ± 19.98	-16.88 ± 19.75	0.010
Nausea/vomiting	0.97 ± 4.00	4.61 ± 12.79	0.109	0.94 ± 5.58	0.52 ± 2.96	0.932	-0.03 ± 4.00	-4.09 ± 12.62	0.047
Pain	29.51 ± 23.17	28.36 ± 22.58	0.781	23.37 ± 21.46	12.67 ± 13.12	0.851	-6.14 ± 18.52	-15.70 ± 14.45	0.014
Dyspnea	12.29 ± 18.14	14.06 ± 20.42	0.792	10.4 ± 17.58	10.06 ± 17.09	0.272	-1.89 ± 19.61	-4.00 ± 21.98	0.633
Insomnia	32.26 ± 29.73	30.21 ± 33.73	0.576	33.26 ± 29.23	15.64 ± 20.73	0.486	1.00 ± 24.96	-14.58 ± 30.36	0.020
Appetite loss	3.77 ± 10.65	8.03 ± 16.67	0.261	4.71 ± 11.72	3.55 ± 9.07	0.827	0.94 ± 12.62	-4.48 ± 17.77	0.251
Constipation	8.51 ± 16.78	11.06 ± 23.04	0.836	7.54 ± 14.06	1.00 ± 5.74	0.400	-0.97 ± 15.01	-10.06 ± 22.77	0.092
Diarrhea	2.83 ± 9.37	7.00 ± 13.70	0.144	6.60 ± 13.39	4.00 ± 10.94	0.827	3.77 ± 10.65	-3.00 ± 15.13	0.038
Financial	18.00 ± 24.68	21.18 ± 31.01	0.939	15.14 ± 20.31	18.15 ± 32.38	0.523	-2.86 ± 20.39	-3.03 ± 24.07	0.810
Global quality of life	71.94 ± 19.47	67.15 ± 11.22	0.319	69.63 ± 17.14	79.55 ± 10.58	0.627	-2.31 ± 19.25	12.39 ± 8.08	0.000

EORTC QLQ-C30, European Organization for Research and Treatment of Cancer Quality of Life Questionnaire Core 30; SD, standard deviation.

*Data were presented as mean ± SD; control group (n = 35); exercise group (n = 33).

^#^P value for difference between groups at baseline or post-intervention.

^&^P value for change between groups from baseline to post-intervention.

#### Changes in sleep quality

**Table 3** summarizes the sleep quality index based on scores of the PSQI. The baseline values for sleep quality did not differ between groups. Overall change of PSQI score decreased by 4.85 in the Baduanjin exercise group compared with 0.34 in the control group (P < 0.01). Significant changes from baseline to post-intervention were also observed for sleep quality (P = 0.001), sleep latency (P = 0.007), sleep duration (P = 0.010), sleep efficiency (P = 0.001), sleep disturbances (P = 0.001), and daytime dysfunction (P = 0.001). Results of the MDC_90_ estimates and the proportion of participants who met the MDC_90_ were summarized in **Supplementary Table S1**.

**Table 3:** Effects of Baduanjin on sleep quality index (PSQI)*.

	Baseline		P^#^	Post-intervention		P^#^	Change		P^&^
	Control	Exercise		Control	Exercise		Control	Exercise	
Subjective sleep quality	1.57 ± 0.81	1.73 ± 0.84	0.455	1.49 ± 0.89	0.91 ± 0.63	0.769	-0.09 ± 0.85	-0.82 ± 0.77	0.001
Sleep latency	1.66 ± 1.11	1.67 ± 1.14	0.965	1.57 ± 1.12	1.00 ± 0.71	0.913	-0.09 ± 0.74	-0.67 ± 0.89	0.007
Sleep duration	1.57 ± 0.85	1.61 ± 0.90	0.870	1.51 ± 0.95	1.06 ± 0.70	0.384	-0.06 ± 0.68	-0.55 ± 0.71	0.010
Sleep efficiency	1.06 ± 1.08	1.15 ± 1.20	0.897	1.29 ± 1.25	0.39 ± 0.56	0.066	0.23 ± 1.00	-0.76 ± 1.06	0.001
Sleep disturbances	1.49 ± 0.61	1.64 ± 0.86	0.508	1.43 ± 0.70	0.82 ± 0.58	0.648	-0.06 ± 0.68	-0.82 ± 0.95	0.001
Use of sleeping medication	0.40 ± 0.77	0.33 ± 0.85	0.375	0.31 ± 0.76	0.15 ± 0.44	0.361	-0.09 ± 0.70	-0.18 ± 0.68	0.566
Daytime dysfunction	1.89 ± 0.99	2.00 ± 1.00	0.607	1.69 ± 0.99	0.94 ± 0.50	0.657	-0.20 ± 0.99	-1.06 ± 0.93	0.001
PSQI score	9.63 ± 3.98	10.12 ± 4.05	0.615	9.29 ± 4.52	5.27 ± 2.14	0.401	-0.34 ± 2.73	-4.85 ± 2.96	0.000

PSQI, Pittsburgh Sleep Quality Index; SD, standard deviation.

*Data were presented as mean ± SD; control group (n = 35); exercise group (n = 33).

^#^P value for difference between groups at baseline or post-intervention.

^&^P value for change between groups from baseline to post-intervention.

### Adverse events

Thirty-three (92%) of the 36 total participants in the Baduanjin exercise group completed the training sessions. There were no major adverse events or complications found during the study.

## Discussion

The objective of this study was to evaluate the effectiveness of the Baduanjin exercise employed in breast cancer patients who were under AI treatment. The data suggest that 12-week Baduanjin exercise significantly increases self-reported quality of life and sleep quality in those participants when compared with those in the control group. In the intragroup analysis, there were significant statistical differences only in the Baduanjin intervention group. Results from this study support the hypothesis that Baduanjin has positive influences on most functioning subscales and global score of quality of life; sleep quality scores were also beneficially changed. AI treatment in breast cancer patients has several serious side effects such as musculoskeletal problems (25), menopausal syndromes (26), and sleep disorders (27), which can lead to low treatment adherence (6) and reduced quality of life (7). It has been consistently shown that the Baduanjin exercise can improve breast cancer patients’ quality of life and sleep quality (22); however, to the best of our knowledge, there are few randomized clinical trials available that evaluated the effect of the Baduanjin training in breast cancer patients under AI treatment.

Several instruments identifying quality of life have been validated for breast cancer survivors, among which the EORTC QLQ-C30 and Functional Assessment of Cancer Therapy-Breast (FACT-B) are most commonly used for the Baduanjin intervention on breast cancer patients (4). The current results of quality of life based on the EORTC QLQ-C30 showed that Baduanjin had obvious beneficial effects on most functioning subscales (physical, role, emotional, and cognitive functioning); in particular, the change of physical functioning in the intervention group increased by 8.48 while it decreased by 3.66 in the control group with significant difference. Notably, the change in fatigue symptom subscales in the Baduanjin group was also significantly lower than that of the control, and the global quality of life score increased by 12.39 compared with 2.31 decrease in the control group. These results reached or exceeded the minimal important difference established in a previous study with patients suffering from advanced cancer (28). Since fatigue is one of the most disturbing adverse reactions in breast cancer patients and can seriously affect the patient’s physical function and quality of life (29, 30), the Baduanjin training-induced decrease of fatigue scores might be one important factor contributing to the improved self-reported quality of life (31). The current observation is in line with recent published studies (14, 18) that focus on the influences of the Baduanjin intervention on physical and psychological health and cognitive function among in women with breast cancer even receiving chemotherapy.

Sleep-related disorders such as insomnia complaints exceed half of AI users who were diagnosed with breast cancer, which is also highly associated with other clinical symptoms including anxiety, depression, and hot flashes (27). In the current study, we observed that the Baduanjin training improved self-reported sleep quality (indicated by lower PSQI scores except for the use of sleeping medication); previous studies also found that Baduanjin significantly improved insomnia measured by PSQI with elevated levels of serum melatonin (32). For breast cancer patients under AI therapy, our findings address the gap and additionally support that the Baduanjin training is an effective intervention strategy leading to improvement of self-reported quality of life.

Even though the biological process contributing to the observed outcome is unclear, inflammatory markers might play a role. Chronic inflammation is present in breast cancer (33) and is considered as a key biological factor causing fatigue and decreased physical function in those patients (34). There is evidence that among women taking AIs, the coexistence of fatigue, insomnia, and arthralgia shared an inflammaotry mechanism (35) and that mind–body interventions (such as Baduanjin) could reduce inflammatory markers (36). Hence, it seems reasonable to speculate that long-term Baduanjin training might suppress inflammatory activities caused by AI treatment; to prove this assumption, further well-designed research is required. Moreover, studies focusing on this effectiveness of the Baduanjin training were encouraged to include physical performance (muscle strength) and physiological parameters (heart rate variability and body composition) as objective measurements, which would add to the evidence derived from questionnaires.

Given that the Baduanjin training is a safe, low-cost, and whole-body intervention that requires the use of multiple muscles and joints and incorporated rhythmic abdominal breathing and meditation (19), our findings support the idea that the Baduanjin training can be an effective intervention strategy in breast cancer patients undergoing AI treatment.

This study has 2 limitations. Firstly, inhibitor-induced arthralgia symptoms were not included as part of the evaluation because not all patients had inhibitor-induced arthralgia, which might lead to underestimation of Baduanjin’s potential benefit. Secondly, we did not collect lifestyle data on the level of physical activity in both groups; it is unclear whether patients were participating in other types of low- to moderate-intensity exercise during the intervention.

## Conclusion

In summary, the current study suggests that 12-week Baduanjin exercise training may be a low-risk, well-tolerated, and safe intervention strategy that leads to improvements of self-reported quality of life and subjective sleep quality in breast cancer patients undergoing AI treatment.

## Data availability statement

The raw data supporting the conclusion of this current study will made available by the corresponding authors without undue reservation.

## Ethics statement

This study involving human participants were reviewed and approved by the Ethics Committee of Guangdong Provincial People’s General Hospital. All participants provided written informed consent. This trial was registered in Clinical Trials.gov and all procedures were in accordance with the principles stated in the declaration of Helsinki.

## Author contributions

JL analyzed the data, drew the graph, and drafted the tables and manuscript. YC conceived and designed the research, performed the research, analyzed the data, contributed to materials and analysis tools. LC performed the research and conduct the Badaunjin exercise program. KW, SW, LW, and BS contributed to conceiving the trial, performing the research, and analyzing the data. MH and XH designed the research, provided assistance, and reviewed the manuscript and tables. All authors contributed to the article and approved the submitted version.

## Funding

This study was supported by grants from the collaborative innovation team of physical activity and health promotion for the Great Bay Area of Guangdong-HongKong-Macau, the Major Science and Technology Projects of Guangdong Province (No. 20130325C), Distinguishing Innovation Project of Department of Education of Guangdong Province (No. 2015 KTSCX080; No. 2016KTSCX070), and the Major International Cooperation Project of Department of Education of Guangdong Province (No. 2014WGJHZ005).

## Acknowledgments

The authors would like to thank Lina Zhao, School of Public Health, Sun Yat-sen University for her assistance with reviewing drafts of the paper.

## Conflict of interest

The authors declare that the research was conducted in the absence of any commercial or financial relationships that could be construed as a potential conflict of interest.

## Publisher’s note

All claims expressed in this article are solely those of the authors and do not necessarily represent those of their affiliated organizations, or those of the publisher, the editors and the reviewers. Any product that may be evaluated in this article, or claim that may be made by its manufacturer, is not guaranteed or endorsed by the publisher.

## References

[1] DeSantis CE Ma J Gaudet MM Newman LA Miller KD Goding Sauer A. Breast cancer statistics, 2019. CA Cancer J Clin (2019) 69(6):438–51. doi: 10.3322/caac.21583 31577379

[2] Burstein HJ Lacchetti C Griggs JJ. Adjuvant endocrine therapy for women with hormone receptor-positive breast cancer: ASCO clinical practice guideline focused update. J Oncol Pract (2019) 15(2):106–7. doi: 10.1200/JOP.18.00617 30523754

[3] Laroche F Perrot S Medkour T Cottu PH Pierga JY Lotz JP. Quality of life and impact of pain in women treated with aromatase inhibitors for breast cancer. a multicenter cohort study. PloS One (2017) 12(11):e0187165. doi: 10.1371/journal.pone.0187165 29117210 PMC5678681

[4] Chopra I Kamal KM. A systematic review of quality of life instruments in long-term breast cancer survivors. Health Qual Life Outcomes (2012) 10:14. doi: 10.1186/1477-7525-10-14 22289425 PMC3280928

[5] Fiorentino L Ancoli-Israel S. Insomnia and its treatment in women with breast cancer. Sleep Med Rev (2006) 10(6):419–29. doi: 10.1016/j.smrv.2006.03.005 PMC2757010 16963293

[6] Verma S Madarnas Y Sehdev S Martin G Bajcar J. Patient adherence to aromatase inhibitor treatment in the adjuvant setting. Curr Oncol (2011) 18 Suppl 1:S3–9. doi: 10.3747/co.v18i0.899 PMC3119895 21698059

[7] Berkowitz MJ Thompson CK Zibecchi LT Lee MK Streja E Berkowitz JS. How patients experience endocrine therapy for breast cancer: an online survey of side effects, adherence, and medical team support. J Cancer Surviv (2021) 15(1):29–39. doi: 10.1007/s11764-020-00908-5 32804353 PMC7430212

[8] Meneses K Azuero A Hassey L McNees P Pisu M. Does economic burden influence quality of life in breast cancer survivors? Gynecol Oncol (2012) 124(3):437–43. doi: 10.1016/j.ygyno.2011.11.038 PMC3278545 22138013

[9] Barron JJ Quimbo R Nikam PT Amonkar MM. Assessing the economic burden of breast cancer in a US managed care population. Breast Cancer Res Treat (2008) 109(2):367–77. doi: 10.1007/s10549-007-9650-4 17674201

[10] Del-Rosal-Jurado A Romero-Galisteo R Trinidad-Fernandez M Gonzalez-Sanchez M Cuesta-Vargas A Ruiz-Munoz M. Therapeutic physical exercise post-treatment in breast cancer: A systematic review of clinical practice guidelines. J Clin Med (2020) 9(4): 1239. doi: 10.3390/jcm9041239 PMC7230832 32344683

[11] Ramirez-Parada K Lopez-Garzon M Sanchez-Rojel C Petric-Guajardo M Alfaro-Barra M Fernandez-Verdejo R. Effect of supervised resistance training on arm volume, quality of life and physical perfomance among women at high risk for breast cancer-related lymphedema: A study protocol for a randomized controlled trial (STRONG-b). Front Oncol (2022) 12:850564. doi: 10.3389/fonc.2022.850564 35299753 PMC8921986

[12] Invernizzi M de Sire A Lippi L Venetis K Sajjadi E Gimigliano F. Impact of rehabilitation on breast cancer related fatigue: A pilot study. Front Oncol (2020) 10:556718. doi: 10.3389/fonc.2020.556718 33194622 PMC7609789

[13] de Sire A Lippi L Ammendolia A Cisari C Venetis K Sajjadi E. Physical exercise with or without whole-body vibration in breast cancer patients suffering from aromatase inhibitor-induced musculoskeletal symptoms: A pilot randomized clinical study. J Pers Med (2021) 11(12): 1369. doi: 10.3390/jpm11121369 34945841 PMC8707128

[14] Ying W Min QW Lei T Na ZX Li L Jing L. The health effects of baduanjin exercise (a type of qigong exercise) in breast cancer survivors: A randomized, controlled, single-blinded trial. Eur J Oncol Nurs (2019) 39:90–7. doi: 10.1016/j.ejon.2019.01.007 30850143

[15] Zou L Pan Z Yeung A Talwar S Wang C Liu Y. A review study on the beneficial effects of baduanjin. J Altern Complement Med (2018) 24(4):324–35. doi: 10.1089/acm.2017.0241 29227709

[16] Koh TC. Baduanjin – an ancient Chinese exercise. Am J Chin Med (1982) 10(1–4):14–21. doi: 10.1142/S0192415X8200004X 7183203

[17] Fang J Zhang L Wu F Ye J Cai S Lian X. The safety of baduanjin exercise: A systematic review. Evid Based Complement Alternat Med (2021) 2021:8867098. doi: 10.1155/2021/8867098 33552220 PMC7847359

[18] Wei XL Yuan RZ Jin YM Li S Wang MY Jiang JT. Effect of baduanjin exercise intervention on cognitive function and quality of life in women with breast cancer receiving chemotherapy: study protocol of a randomized controlled trial. Trials (2021) 22(1):405. doi: 10.1186/s13063-021-05355-w 34147107 PMC8214282

[19] Zou L SasaKi JE Wang H Xiao Z Fang Q Zhang M. A systematic review and meta-analysis baduanjin qigong for health benefits: Randomized controlled trials. Evid Based Complement Alternat Med (2017) 2017:4548706. doi: 10.1155/2017/4548706 28367223 PMC5359459

[20] Zou L Yeung A Quan X Hui SS Hu X Chan JSM. Mindfulness-based baduanjin exercise for depression and anxiety in people with physical or mental illnesses: A systematic review and meta-analysis. Int J Environ Res Public Health (2018) 15(2): 321. doi: 10.3390/ijerph15020321 PMC5858390 29439556

[21] Meng T Hu SF Cheng YQ Ye MN Wang B Wu JJ. Qigong for women with breast cancer: An updated systematic review and meta-analysis. Complement Ther Med (2021) 60:102743. doi: 10.1016/j.ctim.2021.102743 34058368

[22] Kuo CC Wang CC Chang WL Liao TC Chen PE Tung TH. Clinical effects of baduanjin qigong exercise on cancer patients: A systematic review and meta-analysis on randomized controlled trials. Evid Based Complement Alternat Med (2021) 2021:6651238. doi: 10.1155/2021/6651238 33880125 PMC8049783

[23] Health qigong management center of general administration of sport of China: Health qigong-baduanjin. Beijing, China: People’s Sports Publishing House of China (2003).

[24] Kobayashi D Kodera Y Fujiwara M Koike M Nakayama G Nakao A. Assessment of quality of life after gastrectomy using EORTC QLQ-C30 and STO22. World J Surg (2011) 35(2):357–64. doi: 10.1007/s00268-010-0860-2 21104250

[25] Henry NL Giles JT Ang D Mohan M Dadabhoy D Robarge J. Prospective characterization of musculoskeletal symptoms in early stage breast cancer patients treated with aromatase inhibitors. Breast Cancer Res Treat (2008) 111:365–72. doi: 10.1007/s10549-007-9774-6 PMC3081690 17922185

[26] Morales L Neven P Timmerman D Christiaens MR Vergote I Limbergen VE. Acute effects of tamoxifen and third-generation aromatase inhibitors on menopausal symptoms of breast cancer patients. Anticancer Drugs (2004) 15(8):753–60. doi: 10.1097/00001813-200409000-00003 15494636

[27] Desai K Mao JJ Su I Demichele A Li Q Xie SX. Prevalence and risk factors for insomnia among breast cancer patients on aromatase inhibitors. Support Care Cancer (2013) 21(1):43–51. doi: 10.1007/s00520-012-1490-z 22584732 PMC3600410

[28] Bedard G Zeng L Zhang L Lauzon N Holden L Tsao M. Minimal important differences in the EORTC QLQ-C30 in patients with advanced cancer. Asia Pac J Clin Oncol (2014) 10(2):109–17. doi: 10.1111/ajco.12070 23551530

[29] Horneber M Fischer I Dimeo F Ruffer JU Weis J. Cancer-related fatigue: epidemiology, pathogenesis, diagnosis, and treatment. Dtsch Arztebl Int (2012) 109(9):161–71;quiz 72. doi: 10.3238/arztebl.2012.0161 22461866 PMC3314239

[30] Berger AM Gerber LH Mayer DK. Cancer-related fatigue: implications for breast cancer survivors. Cancer (2012) 118(8 Suppl):2261–9. doi: 10.1002/cncr.27475 22488700

[31] Schmidt ME Chang-Claude J Vrieling A Heinz J Flesch-Janys D Steindorf K. Fatigue and quality of life in breast cancer survivors: temporal courses and long-term pattern. J Cancer Surviv (2012) 6(1):11–9. doi: 10.1007/s11764-011-0197-3 22160661

[32] Jiang YH Tan C Yuan S. Baduanjin exercise for insomnia: A systematic review and meta-analysis. Behav Sleep Med (2017), 1–13. doi: 10.1080/15402002.2017.1363042 28777659

[33] Danforth DN. The role of chronic inflammation in the development of breast cancer. Cancers (Basel) (2021) 13(15): 3918. doi: 10.3390/cancers13153918 34359821 PMC8345713

[34] Collado-Hidalgo A Bower JE Ganz PA Cole SW Irwin MR. Inflammatory biomarkers for persistent fatigue in breast cancer survivors. Clin Cancer Res (2006) 12(9):2759–66. doi: 10.1158/1078-0432.CCR-05-2398 16675568

[35] Bauml J Chen L Chen J Boyer J Kalos M Li SQ. Arthralgia among women taking aromatase inhibitors: is there a shared inflammatory mechanism with co-morbid fatigue and insomnia? Breast Cancer Res (2015) 17:89. doi: 10.1186/s13058-015-0599-7 26126656 PMC4504449

[36] Bower JE Irwin MR. Mind-body therapies and control of inflammatory biology: A descriptive review. Brain Behav Immun (2016) 51:1–11. doi: 10.1016/j.bbi.2015.06.012 26116436 PMC4679419

