# Supplementary material for: Baduanjin’s impact on quality of life and sleep quality in breast cancer survivors receiving aromatase inhibitor therapy: a randomized controlled trial
Source: Front Oncol. 2022 Aug 5;12:807531. doi: 10.3389/fonc.2022.807531 (PMC9388824; doi:10.3389/fonc.2022.807531)
Supplement: Supplementary file 1 [file Table_1.docx]

| **Supplementary Table 1 The Minimal Detectable Change and Proportions** **of Participants Who Met the Minimal Detectable Change on Quality of Life and Sleep Quality Index** | | | | | | |
| --- | --- | --- | --- | --- | --- | --- |
|  | MDC_90_ | |  | Proportion Exceeding the MDC_90_ | |  |
|  | Control | Exercise |  | Control | Exercise |  |
| Quality of Life |  |  |  |  |  |  |
| Physical functioning | 5.41 | 5.35 |  | 62.9% | 69.7% |  |
| Role functioning | 5.96 | 6.45 |  | 31.4% | 30.3% |  |
| Emotional functioning | 5.75 | 6.77 |  | 62.9% | 72.7% |  |
| Cognitive functioning | 6.46 | 7.35 |  | 42.9% | 66.7% |  |
| Social functioning | 8.24 | 9.85 |  | 48.6% | 60.6% |  |
| Fatigue | 9.73 | 6.75 |  | 85.7% | 69.7% |  |
| Nausea/vomiting | 1.12 | 5.05 |  | 5.7% | 12.1% |  |
| Pain | 9.21 | 7.44 |  | 54.3% | 48.5% |  |
| Dyspnea | 7.13 | 8.17 |  | 34.3% | 42.4% |  |
| Insomnia | 10.67 | 12.53 |  | 37.1% | 42.4% |  |
| Appetite loss | 3.64 | 6.67 |  | 14.3% | 24.2% |  |
| Constipation | 6.33 | 6.44 |  | 20.0% | 21.2% |  |
| Diarrhea | 4.14 | 5.17 |  | 11.4% | 21.2% |  |
| Financial | 9.56 | 12.07 |  | 31.4% | 24.2% |  |
| Global quality of life | 7.14 | 6.33 |  | 74.3% | 66.7% |  |
| Sleep Quality Index |  |  |  |  |  |  |
| Subjective sleep quality | 0.27 | 0.33 |  | 54.3% | 48.5% |  |
| Sleep latency | 0.44 | 0.46 |  | 45.7% | 39.4% |  |
| Sleep duration | 0.37 | 0.38 |  | 40.0% | 42.4% |  |
| Sleep efficiency | 0.44 | 0.43 |  | 51.4% | 48.5% |  |
| Sleep disturbances | 0.24 | 0.19 |  | 34.3% | 45.5% |  |
| Use of sleeping medication | 0.30 | 0.34 |  | 31.4% | 9.1% |  |
| Daytime dysfunction | 0.41 | 0.38 |  | 60.0% | 45.5% |  |
| PSQI score | 1.54 | 1.66 |  | 57.1% | 63.6% |  |
| Abbreviations: MDC_90_, minimal detectable change at 90% confidence; PSQI, Pittsburgh sleep quality index. | | | | | | |
